# Effectiveness of Stem Cell Therapies in Improving Clinical Outcomes in Patients With Heart Failure

**DOI:** 10.7759/cureus.17236

**Published:** 2021-08-16

**Authors:** Nitin Bhawnani, Aarthi Ethirajulu, Almothana Alkasabera, Chike B Onyali, Comfort Anim-Koranteng, Hira E Shah, Jihan A Mostafa

**Affiliations:** 1 Medicine, California Institute of Behavioral Neurosciences & Psychology, Fairfield, USA; 2 Internal Medicine, California Institute of Behavioral Neurosciences & Psychology, Fairfield, USA; 3 General Medicine, California Institute of Behavioral Neurosciences & Psychology, Fairfield, USA

**Keywords:** heart failure outcomes, stem cell, clinical outcomes, heart failure, stem cell therapy effectiveness

## Abstract

Heart failure (HF), continuing to be a notable cause of morbidity and mortality worldwide, also is a noteworthy economic burden to the patients. Current medical management of HF has poor efficacy to completely arrest or reverse the progression to end-stage disease. As the option of cardiac transplantation remains limited to few patients, the stem cell approach continues to be a promising one in developing a novel therapy in the treatment of HF.

This review attempts to discuss and compare the outcomes of numerous clinical trials that involved treatment of HF of variable etiologies with stem cells of numerous lineages such as bone marrow-derived cells (BMCs), mesenchymal stem cells (MSCs), cardiosphere derived progenitor cells (CDCs), etc. We reviewed articles and randomized controlled trials (RCT) that used stem cells to treat heart failure. The articles and RCT studies were obtained through a search on PubMed and Medline databases and performed using regular and medical subject heading (MeSH) keyword search strategy. A total of 17 trial-based studies, along with other articles that met the aim of the review, were selected. A discussion of the findings from major clinical trials such as the C-CURE, CHART-1, POSEIDON, POSEIDON-DCM, TAC-HFT, and other small scale trials highlights the change in functional and mechanical parameters of HF, namely, left ventricular ejection fraction (LVEF), end-diastolic volume (EDV), end-systolic volume (ESV), 6-minute walking test distance (6MWTD), N-terminal pro-B-type natriuretic peptide (NT-proBNP) levels and assessment of New York heart association (NYHA) class of heart failure, and Minnesota Living with Heart Failure Questionnaire (MLHFQ) score to reflect improvement in quality of life (QoL) of patients.

Out of the studies analyzed, the majority reported significant improvements in at least two of the parameters mentioned above. However, more phase three randomized trials are required to compare the efficacy of multiple lineages of stem cells, factoring in molecular and dosage factors to develop a standardized therapy.

## Introduction and background

Despite the recent advances in medical therapy, Heart Failure (HF) remains a significant cause of mortality and morbidity worldwide. Not only does it affect the quality of life of the patients, but it also poses an economic burden. In the USA, the total healthcare costs for patients diagnosed with HF are projected to rise from US$20.9 billion in 2012 to $53.1 billion by 2030, due to both, increase in the prevalence of HF in the population as well as an increase in the cost of care due to inflation [1].

Current therapies to manage HF include combined drug therapies, left ventricular assist devices (LVAD), cardiac resynchronization therapy (CRT), and ultimately cardiac transplantation for end-stage disease [2,3,4]. However, none of these therapies are efficacious in arresting the loss of cardiomyocyte function and development of fibrosis in a failing heart, and the therapeutic option of cardiac transplantation remains unavailable to many due to the large gap between available donors and eligible recipients, further complicated by the need of long-term immunosuppression needed after a transplant.

Evidence has emerged that suggests the possibility of regeneration of myocardial tissue using cardiac stem cells, based upon which numerous clinical trials are probing the use of stem cells from multiple origins as a therapeutic alternative in HF [5]. Available cell types that have the potential to replace lost myocardium include mesenchymal stem cells (MSCs), bone-marrow-derived cells (BMCs), embryonic stem cells (ESCs), cardiac progenitor cells (CPCs), skeletal myoblasts, and hematopoietic stem cells (HSCs) [6,7,8].

Multiple studies conducted in the last decade address the use of stem cells in patients of HF due to variable etiologies. The majority of them differ in types of stem cells, their dosage, and route of administration used in the trial.

While older studies failed to demonstrate any significant clinical improvement in parameters such as left ventricular ejection fraction (LVEF), end-diastolic volume (EDV), recently published studies have shown improvement of cardiac function following stem cell therapy in HF patients [9-15].

Across all age groups and categories of HF based on ejection fraction (EF) (HF with preserved, borderline, or reduced EF), five-year survival remains poor with high rates of hospital readmission and adverse cardiovascular events, prompting the need of developing a novel cell-based therapy for HF [16].

This literature review will focus on assessing the clinical outcomes in HF patients from numerous clinical trials, following stem cell therapies, specifically analyzing functional and mechanical factors such as LVEF, EDV, end-systolic volume (ESV), New York Heart Association (NYHA) class Minnesota Living with Heart Failure Questionnaire (MLHFQ), 6-minute walking test distance (6MWTD), and N-terminal pro-B-type natriuretic peptide (NT-proBNP) levels. Furthermore, we will discuss the efficacy and potential of various cell types, based on available data and evidence, in the development of a novel therapy to treat and potentially devise a cure for HF.

## Review

How effective are mesenchymal stem cells (MSCs) in HF?

The potential use of MSCs in heart failure stems from the evidence that suggests that they can differentiate into cardiomyocytes in vivo [17]. The Cardiopoietic stem Cell therapy in heart failURE (C-CURE) trial was a multicenter, randomized trial. Conducted on patients with ischemic heart failure, findings from the C-CURE trial published in 2013 include improvement of LVEF from 27.5 ± 1.0% to 34.5 ± 1.1% in the test group that received MSC in addition to drug therapy (standard care) as compared to the control group that received standard care alone (from 27.8 ± 2.0% to 28.0 ± 1.8%), which was statistically significant (p<0.0001) [18]. It further reported improved distance on 6MWT, also noting significant reduction in LVESV (-24.8 ± 3.0 ml vs. -8.8 ± 3.9 ml, p < 0.001). The trial reported no cardiac or systemic toxic effects as a result of stem cell therapy, establishing a healthy safety profile for trials with similar MSCs [18].

Zhao et al. conducted a trial using umbilical cord MSCs publishing their work in 2015, comparing control (n=29) and treatment (n=30) at one and six months after MSC transplantation [19]. Reduction in mortality between control and treatment groups was statistically significant (p<0.05) at follow up with seven deaths in the control group and two deaths in the treatment group. They also reported a significant fall in NT-proBNP level at one month (p<0.05) and after six months (p<0.01) after the MSC transplant. Similar to the C-CURE trial (17), there was a statistically significant improvement in LVEF (p<0.01), 6MWT (p<0.01) at both one and six months follow-up. However, the difference in readmission rate was not statistically significant [19].

Tompkins et al., in 2018, conducted a post hoc analysis, mainly comparing the efficacy of MSC in ischemic cardiomyopathy (ICM) and dilated cardiomyopathy (DCM) [11]. They noted a 7% increase in LVEF in the DCM patient group but no significant change of EF in the ICM group at follow-up. A significant finding was that DCM patients had improvement in parameters of LVEF and ESV primarily, suggesting recovery of systolic function, while ICM patients had better results with respect to cardiac remodeling, suggested by improvement in EDV, sphericity index, and end-diastolic mass measurements at follow-up. Both cohorts were found to have better functional performance and output indicated by a reduction in NYHA class and better performance in 6MWTD [11]. As noted in the study, this finding points to the fact that MSC and potentially other stem cell therapies may still improve outcomes in HF, regardless of improvement in parameters such as LVEF.

MSCs prove to be a promising option in developing stem cell therapy for HF of different etiologies, as the majority of the studies point to its beneficial role in arresting the disease process as well as improving cardiac performance. However, the etiology of heart failure may significantly affect the therapeutic efficacy of MSCs, as indicated by findings from Tompkins et al.'s analysis [11].

Tompkins et al., with a post hoc analysis that includes three different randomized, blinded clinical trials, provide an assessment of data from a total of 123 patients (n=50 for autologous MSC; n=33 for allogenous MSC; n=19 for BMC transplant; and n=21 for the placebo group) from Transendocardial Autologous Mesenchymal Stem Cells and Mononuclear Bone Marrow Cells in Ischemic Heart Failure Trial (TAC-HFT), The Percutaneous Stem Cell Injection Delivery Effects on Neomyogenesis Pilot Study (POSEIDON study), and Percutaneous Stem Cell Injection Delivery Effects on Neomyogenesis in Dilated Cardiomyopathy (POSEIDON-DCM) trials proving to be one of the most reliable data on the outcome of MSC therapy in HF [11,20-22]. Albeit with a smaller cohort of 36 patients (n=15 for control and n=21 for treatment group), the C-CURE trial brought forth the clinical evidence of superior benefit and improvement after using lineage-specific stem cells in HF, as established in the study by Behfar et al. that found that guided cardiopoiesis has the potential to enhance the therapeutic efficacy of stem cells [23]. Zhao et al.'s study provides no information on blinding and the process of randomization of the trial and is thus more prone to a bias in findings and results [19].

Comparison of Efficacy of Allogenous and Autologous Groups of MSCs

Hare et al. conducted the POSEIDON-DCM trial that compared the efficacy of allogenic and autologous MSC in non-ischemic dilated cardiomyopathy (NIDCM) patients. With 18 patients in the allogeneic MSC group and 16 patients in the autologous MSC group, the trial assessed the outcomes at 30 days, three-, six-, and twelve months after treatment [22].

Major findings included a statistically significant low all-cause rehospitalization rate in the allo-group vs. the auto-group (p=0.0447). EF assessment at 12 months revealed a significant improvement in the allo-group (p=0.004) but no significant change in the auto-group (p=0.49).

Similarly, 6MWT results demonstrated a significant increase in the allo-group but not in the auto-group (p=0.04 vs. p=0.71). However, there was no significant reduction in EDV and ESV in either of the populations [22].

A beneficially preferential response to allogeneic MSC reflects the need to investigate and compare the potential and efficacy of major cell lineages that are being researched to cure or reverse HF.

The POSEIDON-DCM trial is the only trial reviewed here that compares and assesses the benefits achieved by autologous vs. the allogenous group of MSC’s [22]. With significantly different findings in these groups, trials similar to the POSEIDON-DCM trial are needed to compare two or more different cell lineages and groups of BMCs, MSCs, CPCs, ESCs, etc., to find the most effective stem cell approach for HF.

How effective are bone-marrow-derived cells (BMCs) in HF?

Martino et al. in 2015 used intracoronary injections of autologous bone marrow mononuclear cells (BMNCs) in patients with NIDCM and LVEF<35% comparing them against a group that received a placebo [24]. They found no statistical improvements in the mechanical parameters (EF, EDV, ESV) or in the mortality rate. Clinical parameters (NYHA class and MLHFQ) also failed to show any improvement in this trial [24].

Hamshere et al. conducted a randomized, placebo-controlled trial dividing the cohorts into four different groups [14]. The study aimed at researching the efficacy of Intracoronary delivered BMCs in adjuvant to granulocyte-colony stimulating factor (G-CSF). The four groups received only one out of these: placebo, G-CSF (Granulocyte-Colony Stimulating Factor), G-CSF+IC (intracoronary) Serum, and G-CSF+IC-BMCs. At three months and one-year follow-ups, the treatment group that received G-CSF+IC-BMC showed significant improvement with a 5.37% increase in the LVEF and reduction of NT-proBNP levels, NYHA class, and improved QoL. LVEF showed no significant improvement in any of the other three groups [14].

Frljak et al., in 2018, studied the effect of a cluster of differentiation (CD) 34+ BMC in improving the right ventricle (RV) functioning in patients suffering from NIDCM [25]. The cells were extracted after the treatment group received G-CSF. The findings consisted of improved viability of interventricular septum resulting in improved RV performance assessed via tricuspid annular plane systolic excursion (TAPSE), which was the primary endpoint of the study, in addition to improvements in LVEF, EDV, and 6MWTD (secondary endpoints). This study is of paramount importance in developing therapies for patients with biventricular failure.

Soestina et al. published the results of a trial that investigated the use of CD133+ BMCs implanted trans-epicardially and trans-septally during the coronary artery bypass graft (CABG) procedure in order to improve cardiac function [15]. They reported a significant increase (p=0.04) in EF at six months post-CABG in the group that received CD133+ BMCs against the group that received CABG alone [15].

Although the earlier study by Martino et al. failed to demonstrate the efficacy of BMC therapy in HF patients, newer trials have shown that BMCs of different lineages adjuvant to G-CSF [14] or extracted after pre-treatment with G-CSF [25] were more likely to produce promising results. Additionally, findings of Frljak et al. suggest that these cells can benefit patients with biventricular failure as well [25]. However, the lineage of cells, dosage, and route of administration (ROA) being different in all of the above-mentioned trials make it difficult to conclude the efficacy of BMC's concretely. More trials with higher cohort populations and similar cell lineage, dose, and ROA are needed.

Martino et al. conducted a randomized, double-blind, placebo-controlled clinical trial of a total of 160 patients (n=82 for the treatment group and n=78 for the placebo group) [24]. Only 61 patients were analyzed post stem cell therapy, as 21 were lost during follow-up in the treatment group. 24 patients from the placebo group were also lost during follow-up. A total of 115 patients of the study underwent complete follow-up and analysis. With no significant improvement in outcomes studied on a relatively larger cohort of 115 patients, this trial further necessitates the need to thoroughly investigate the efficacy of autologous BMCs in HF due to NIDCM and probably even research other available bone marrow cell lineages. Hamshere et al., in a phase-2, randomized, double-blind placebo-controlled trial of a total of 60 patients divided into three treatment groups and one placebo group, provides a detailed assessment and comparison of BMC therapy with and without G-CSF administration [14]. Favorable findings in the treatment group bring to light a new research area of studying stem cell therapies with concomitant administration of G-CSF and other colony-stimulating factors as well. Soestina et al., with a smaller cohort of 26 patients in a single-blind randomized control trial that used CD133+ cells, brings forward the importance of researching lineage-specific BMC as it showed a significant and favorable outcome in HF [15]. Frljak et al. represented the sub-study of a larger randomized study. Similar to Soestina et al., they also used lineage-specific BMC's, which were CD34+ but had a larger cohort of 60 patients (n=30 for the treatment group and n=30 for the placebo group). It is one of the few studies to have studied RV function and improvement in addition to left ventricular parameters and hence an important one with respect to developing a therapy for biventricular failure [25].

The emerging potential of cardiosphere-derived cells (CDCs) in HF

Cardiosphere-derived cells (CDCs) represent a heterogeneous group of stem cells that express both hematopoietic and mesenchymal antigens [26].

Sano et al., in an integrated cohort study of 101 patients, evaluated the potential efficacy of CDCs in HF patients. At two-year follow-up with stage-specific ventricular function assessment via two-dimensional echocardiography, the CDC-treated group of patients showed significant improvement in ventricular function (stage 2: +8.4±10.0% versus +1.6±6.4%, P=0.03; stage 3: +7.9±7.5% versus −1.1±5.5%, P<0.001), and reduced risk of adverse complications as compared to the control group (p=0.013). A notable finding also reported was that CDC infusion did reduce mortality (p=0.038) and late complications (p<0.05) in patients with HF with reduced EF but failed to do so in HF with preserved EF. [27]

In 2020, a publication by Makkar et al. researched potential myocardial regeneration after intracoronary infusion of autologous cardiosphere derived/cardiac progenitor cells (CDCs), enrolling patients four to twelve weeks after myocardial infarction that had an EF of less than 45% and left ventricular scar size of more than 15%. Although prematurely stopped, the data available at six months after infusion of CDCs revealed a statistically significant reduction in LVEDV (p=0.02), LVESV (p=0.02), and NT-proBNP levels (p=0.02). But there was no statistically significant change in scar size as compared against the placebo group [28].

CDC infusion in carefully selected patients does demonstrate a disease-modifying effect that can help arrest the progression of left ventricular dysfunction, as indicated by the reduction in end-diastolic and end-systolic volumes.

With a larger cohort of a total of 134 patients (n=90 for the treatment group and n=44 for the placebo group), this is one of the few trials that used CDCs for stem cell therapeutic intervention. The favorable findings in this multicenter, randomized, double-blinded, placebo-controlled trial represent a positive progression towards achieving the goal of developing a successful stem cell therapy for HF.

Current evidence of the efficacy of CDCs is limited by the fewer number of clinical trials conducted with the use of CDCs.

Table 1 briefly summarizes the outcomes and results of numerous clinical trials and studies pertaining to the use of MSC, bone-marrow derived stem cells (BSC), and CDC therapies in HF.

**Table 1:** Summary of major MSC, BSC, and CDC therapies in heart failure patients Abbreviations: MSC: mesenchymal stem cells, CDC: cardiosphere-derived cells, BSC: bone-marrow derived stem cells, BMNC: bone-marrow mononuclear cells, UC-MSC: umbilical cord-derived mesenchymal stem cells, BMC: bone-marrow-derived cells CD: cluster of differentiation, HF: heart failure EF: ejection fraction, LVEF: left ventricular ejection fraction, ESV: end-systolic volume, EDV: end-diastolic volume, 6MWTD: 6-minute walking test distance, NYHA: New York Heart Association, NT-proBNP: N-terminal pro-B-type natriuretic peptide, RV: right ventricle, NIDCM: non-ischemic dilated cardiomyopathy, CAD: coronary artery disease.

Study	Types of cells	No of patients that received stem cell therapy	Major baseline characteristics	Outcomes	Year of study
Hare et al., [21]	Allogenic MSC Autologous MSC	18 - Allogenic 16- autologous	LVEF < 40% NIDCM	Greater increase in EF in allogenic group vs the autologous group.	2012
Bartunek et al., [18]	MSC	21	LVEF: 15-40%	Increased LVEF ; Decreased ESV; Decreased EDV; Improved 6MWTD.	2013
Martino et al., [24]	BMNC	61	LVEF < 35% and NIDCM	No significant improvement in LVEF, ESV and EDV.	2015
Hamshere et al., [14]	BMC	30	LVEF < 45% and DCM	Increased LVEF; Reduction in NYHA class.	2015
Zhao et al., [19]	UC-MSC	30	LVEF < 35% NYHA III-IV HF	Increased LVEF; Increased 6MWTD; Reduced Mortality; Reduction in NT-proBNP level.	2015
Frljak et al., [25]	CD 34+ Cells	30	LVEF < 40% NIDCM	Increased LVEF; Reduced NT-proBNP; Improved 6MWTD; Improved RV function.	2018
Soetisna et al., [15]	CD 133+ BMC	13	LVEF < 35% and CAD	Increased LVEF; Improved 6MWTD; Decreased Scar size proportion.	2020
Makkar et al., [28]	Cardiac Progenitor Cells	90	LVEF < 45%	Reduced EDV; Reduced ESV; Reduced NT-proBNP; No change in scar size.	2020

How effective are skeletal myoblasts or muscle-derived stem cells in HF?

Duckers et al. conducted a phase-2 randomized, open-label trial that aimed to evaluate the efficacy of autologous skeletal myoblasts in HF patients [29]. The SEISMIC trial enrolled 40 patients (n=26 in the treatment group and n=14 in the control group) and defined 6MWTD, LVEF, and change in NYHA class as efficacy endpoints. Six months after the transplantation of the cells, 6MWTD improved in the treatment group, whereas there was no improvement in the control group. However, there was no statistically significant improvement in LVEF in either of the groups [29].

Sawa et al. in 2015 enrolled seven patients in a phase-2, multicenter study to test the efficacy of autologous skeletal myoblast sheets (TCD-51073) in patients suffering from severe chronic HF [30]. At 26-weeks follow-up, the 6MWTD significantly improved from 410.1±136.1 m before the transplant to 455.4±103.7 m after the transplant. Six among the seven enrolled patients demonstrated an improvement of at least one class in the NYHA measurement scale [30].

Gwizdala et al. in 2017 investigated the safety and efficacy of muscle-derived stem/progenitor cells (MDS/PCs) that were modified with connexin-43 (Cx-43) gene for treatment of advanced HF [31]. A total of 13 patients with NYHA class II-III HF were enrolled and treated with an injection of Cx-43-MDS/PCs. There was a statistically significant improvement in NYHA class from 3 ± 0 before the treatment and 1.8 ± 0.7 after the treatment (p=0.003). They also noted improvement in exercise capacity, LVEF, and LVEDV in the patients. No deaths or significant arrhythmias were noted, establishing the safety profile of Cx-43 treated MDS/PCs [31].

Limited by the number of trials conducted and smaller patient populations in most of them, the efficacy of skeletal myoblasts or muscle-derived stem cells is yet to be clearly established. Gwizdala et al.’s study provides promising data regarding the safety and efficacy of gene-modified muscle-derived stem cells and does establish the base for future studies for gene-modified cell lineages to be tried in HF.

Table 2 provides an overview of the major findings from trials that used muscle-derived stem cells or skeletal myoblasts for the treatment of HF.

**Table 2:** Summary of trials using muscle-derived stem cells or skeletal myoblasts for treatment of HF Abbreviations: HF: Heart Failure, LVEF: Left Ventricular Ejection Fraction, ESV: end-systolic volume, EDV: end-diastolic volume, 6MWTD: 6-minute walking test distance, NYHA: New York Heart Association.

Study	Types of cell	No of patients that received stem cell therapy	MAJOR BASELINE CHARACTERISTICS	OUTCOMES	Year of study
Duckers et al., [29]	Autologous skeletal myoblasts	26	NYHA class II-III HF; LVEF: 20-40%	6MWTD increased No improvement in LVEF	2011
Sawa et al., [30]	Autologous skeletal myoblasts	7	NYHA class III-IV HF; LVEF <35%	Improvement in NYHA class; Improvement in 6MWTD.	2015
Gwizdala et al., [31]	Muscle derived cells modified with gene Connexin-43	13	NYHA class II-IV HF; LVEF < 40%	Improvement in NYHA class; Improvement in LVEF, EDV, and ESV; Improvement in exercise capacity.	2017

Limitations

This literature review has numerous limitations. There were no specific inclusion or exclusion criteria to select the studies. The search was limited to Medline/PUBMED database. The analysis does not consider the difference in dosage and route of administration of stem cells which can affect the outcome of the study. The studies were neither segregated based on the etiology of HF, such as ischemic or cardiomyopathic, nor based on the systolic or diastolic category of failure. It only focused on comparing the results and outcomes based on functional and mechanical parameters of HF. However, not all the studies selected had the same efficacy endpoints and comparison was made only on the basis of improvements in major parameters.

## Conclusions

This literature review of clinical trials and studies of stem cell therapies in HF focused on finding the improvement in functional and mechanical parameters of HF such as LVEF, 6MWTD, LVEDV, LVESV, QoL, and reduction in NYHA class and MLHFQ improvement, thus assessing if stem cell therapy improves outcomes or not. Out of the studies analyzed, most used BMCs or MSCs, and favorable results were found in all except one, with the majority of the studies reporting significant improvement in the above-mentioned parameters. More phase three, randomized, double-blind, placebo-controlled trials are required with both lineage-specific and non-specific cells, and trials that pitch and compare the efficacy of different cells against each other are required to develop a standardized novel therapy for HF. Furthermore, the effect of molecular factors such as CD factor differentiation on the efficacy of stem cell therapy needs to be investigated.
